# Supplementary material for: Empirical methods for controlling false positives and estimating confidence in ChIP-Seq peaks
Source: BMC Bioinformatics. 2008 Dec 5;9:523. doi: 10.1186/1471-2105-9-523 (PMC2628906; doi:10.1186/1471-2105-9-523)
Supplement: Additional File 1 — A variety of html documents from the USeq web site detailing the available applications, their best usage, output file type descriptions, command line menus, etc. [file 1471-2105-9-523-S1.zip › USeqUserGuides/JavaDoc.zip!/JavaDoc/bioroot/class-use/Feature.html]

Uses of Class bioroot.Feature


|  |  |  |
| --- | --- | --- |
| **Overview** \| **Package** \| **Class** \| **Use** \| **Tree** \| **Deprecated** \| **Index** \| **Help** | |  |
| PREV   NEXT | **FRAMES**    **NO FRAMES**     **All Classes** |  |


---


## **Uses of Class bioroot.Feature**

| Packages that use Feature | |
| --- | --- |
| **bioroot** |  |
| **bioroot.antibody** |  |

Uses of Feature in bioroot

| Methods in bioroot that return Feature | |
| --- | --- |
| `Feature[]` | `Preference.getFeatures()` |
| `static Feature[]` | `Feature.makeFeatures(java.lang.String concat)`             Makes a Feature[] from a String concatinate, the thing that is actually stored in the database. |
| `static Feature[]` | `Util.makeFeatures(java.lang.String[] stringFeatures)`             Use to make a default Feature[]. |

| Methods in bioroot with parameters of type Feature | |
| --- | --- |
| `static java.lang.String` | `Feature.createFeatureConcat(Feature[] features)`             Creates a String concatinate from a Feature[] for storage in the database. |
| `static java.lang.String` | `Feature.fetchCheckBoxNumberTableRows(Feature[] features, int numColumns)` |
| `static java.lang.String` | `Archive.fetchTabbedTableColumnRow(Feature[] features)`             Returns a table header row using the Preferece obj |
| `static java.lang.String[]` | `Feature.getFeatureNames(Feature[] features)` |
| `void` | `Preference.setFeatures(Feature[] features)` |

Uses of Feature in bioroot.antibody

| Methods in bioroot.antibody that return Feature | |
| --- | --- |
| `static Feature[][]` | `AntibodyBase.splitAntibodyFeatures(Feature[] all)`             Returns arrays of Features where the F[0] = antibody, F[1]=abPrep, F[2]=useDil specific features. |

| Methods in bioroot.antibody with parameters of type Feature | |
| --- | --- |
| `static Feature[][]` | `AntibodyBase.splitAntibodyFeatures(Feature[] all)`             Returns arrays of Features where the F[0] = antibody, F[1]=abPrep, F[2]=useDil specific features. |

---


|  |  |  |
| --- | --- | --- |
| **Overview** \| **Package** \| **Class** \| **Use** \| **Tree** \| **Deprecated** \| **Index** \| **Help** | |  |
| PREV   NEXT | **FRAMES**    **NO FRAMES**     **All Classes** |  |


---
